# Molecular characterization of a rare heterozygous *APOA5* variant in a Chinese family with moderate hypertriglyceridemia

**DOI:** 10.3389/fgene.2026.1796970

**Published:** 2026-06-02

**Authors:** Hongxia Liu, Xiao Yuan, Jie Wang, Yuande Mao, Jinxi Wang, Dongli Wang, Fang Tian, Yili Yang, Bo Xiang

**Affiliations:** 1 The First Affiliated Hospital of Jishou University, Jishou, Hunan, China; 2 China Regional Research Centre, International Centre for Genetic Engineering and Biotechnology, Taizhou, Jiangsu, China; 3 Guangzhou BroadBio Technology Co., Ltd., Guangzhou, Guangdong, China; 4 The First Affiliated Hospital of Hunan University of Chinese Medicine, Changsha, Hunan, China

**Keywords:** APOA5, genetic testing, hypertriglyceridemia, p.R223C, WES

## Abstract

**Background:**

The development of hypertriglyceridemia (HTG) can be attributed to either a monogenic or a polygenic etiologic basis, and the understanding of this molecular basis is incomplete. APOA5 plays a critical role in triglyceride (TG) metabolism, and APOA5 deficiency is a recognized cause of HTG. However, the effects of rare *APOA5* variants observed only in isolated cases are often difficult to establish conclusively. This study aims to find the genetic cause of moderate HTG in a Chinese family, and conduct preliminary *in silico* verification.

**Methods:**

Eight family members received biochemical testing, and genetic testing based on whole-exome sequencing (WES). Basic information including body mass index (BMI), medical history, prescription for TG management, and smoking and drinking habits was recorded. Comprehensive residue conservation analysis and computational simulation of protein structure stability were performed to measure the impact of the assumptive causal variant.

**Results:**

A rare heterozygous *APOA5* variant (p.R223C) was identified. Specifically, six family members who carried the variant had substantially higher fasting plasma TG level than the admitted threshold (1.7 mmol/L) with the highest of 4.96 mmol/L, while a non-carrier in this family was normal in TG. The p. R223C variant was absent from ClinVar and gnomAD databases. Besides, *in silico* predictions results supported the variant’s potential deleteriousness.

**Conclusion:**

This study presents a familial case of moderate HTG associated with a rare *APOA5* variant, which is classified as Likely Pathogenic (LP) according to the ACMG/AMP guideline. The real effect of this variant requires further investigation via biochemical or cell-based studies.

## Introduction

Hypertriglyceridemia (HTG) is characterized by elevated level of triglyceride (TG) in the blood (>1.7 mmol/L) (Parhofer et al., 2019; Kiss et al., 2023), resulting from an imbalance between the production and clearance of TG-rich lipoproteins, such as chylomicrons and very-low-density lipoproteins (VLDLs) (Packard et al., 2020). According to the clinical practice guideline of the Endocrine Society, HTG is further classified as mild (1.7–2.3 mmol/L), moderate (2.3–11.2 mmol/L), severe (11.2–22.4 mmol/L) and very severe (≥22.4 mmol/L) (Berglund et al., 2012). It has been estimated that HTG affects approximately 10% of the global adult population (Carrasquilla and Christiansen, 2021). Affected individuals often exhibit elevated total cholesterol, decreased high-density lipoprotein cholesterol (HDL-C), and normal to low low-density lipoprotein cholesterol (LDL-C) (Parhofer et al., 2019). HTG is also associated with development of atherosclerotic cardiovascular disease, increased risk of pancreatitis (Wolska et al., 2020; Dron and Hegele, 2020), and metabolic disorders such as obesity and type 2 diabetes (Case et al., 2019). Therefore, it is a significant challenge for the health care system.

While metabolic conditions, lifestyle, and certain medical conditions all cause or exacerbate HTG, genetic factors usually play an essential role in the development of this disease (Packard et al., 2020; Dron and Hegele, 2020). HTG can arise from either monogenic or polygenic causes and the understanding of the molecular mechanism is not sufficient yet (Carrasquilla and Christiansen, 2021; Alves et al., 2024). The current viewpoint holds that clinically relevant abnormalities of plasma TG levels typically require a polygenic foundation (Hegele et al., 2014; Dron et al., 2019; Musambil et al., 2020; Dron et al., 2020). Nevertheless, rare variants in genes influencing lipoprotein lipase (LPL) maturation and function (*LPL*, *APOC2*, *APOA5*, *GPIHBP1*, *LMF1*) may lead to monogenic familial HTG (Carrasquilla and Christiansen, 2021; Ramasamy, 2016). And it is believed that identifying these specific mutations and their mechanisms of action will greatly help the precision management and treatment of HTG. APOA5 (Apolipoprotein A5) is a 366-amino acids (AAs) minor apolipoprotein mainly synthesized in the liver, and it increases the activity of LPL, which hydrolyzes TGs in chylomicrons and VLDL from circulation (Guardiola and Ribalta, 2017). Thus, APOA5 acts as a potent regulator of plasma TG levels. In fact, it has been shown that APOA5 deficient mice developed HTG with reduced capillary LPL levels (Yang et al., 2024; Yang et al., 2023). Further, Cakmak et al. identified eight distinct *APOA5* variants in 10 Turkish patients with severe HTG and most of the variants were of uncertain significance. Puerto-Baracaldo et al. reported 17 *APOA5* variants among Colombian HTG individuals, including three novel variants (Puerto-Baracaldo et al., 2024). Using expanding genetic sequencing including 15 TG-related genes, Jin et al. linked the presence of rare *APOA5* variants to severe HTG in a cohort of 103 patients (Jin et al., 2018). Liu et al. identified six pathogenic *APOA5* variants (p.S35N, p. D167V, p. G185C, p. K188I, p. R223C, p. H182fs) correlating with severe HTG in 163 Chinese patients with hyperlipidemic acute pancreatitis (Liu et al., 2024). With the exception of p. G185C, each variant was detected in a single patient.

This study reports a Chinese family harboring a rare heterozygous *APOA5* variant (p.R223C) associated with moderate HTG. To our knowledge, this is the first identification of the variant in a family with HTG. In our study, basic clinical information as well as results of biochemical testing and genetic testing, were documented for preliminary diagnosis. Moreover, a comprehensive residue conservation analysis was performed across 50 species (including 30 mammals) to assess the substitutability of the native arginine at position 223 in APOA5, and the impact to the protein structure stability change upon p. R223C was also simulated. Both results supported the perniciousness of the variant, and taking together our study introduces a family-based evidence for a rare *APOA5* variant involved with familial HTG, which could provide some references and clues for the molecular diagnosis and pathogenesis research on this illness.

## Materials and methods

### Biochemical testing

Venous blood samples were collected from participants after a minimum 12-h fast and 72 h of alcohol abstinence. The blood contents (including LDL-C) were directly measured using cobas® 8,000 modular analyzer series (Roche Diagnostics) following the manufacturer’s standard protocol.

### Genetic testing

EDTA-anticoagulated venous blood was collected from subjects and genomic DNA was extracted from the blood using QIAamp DNA Blood Mini Kit. Exome enrichment experiment was conducted with xGen™ Exome Research Panel v2 kit and the captured exome was then sequenced by the Illumina NovaSeq 6,000 platform. Output reads were preprocessed via fastp (Chen et al., 2018) and then aligned to the human reference genome (hg19) via BWA (Li and Durbin, 2009). The GATK Best Practices recommendations (Van der Auwera et al., 2013) was executed for the variants calling procedure. Called variants were annotated through ANNOVAR (Wang et al., 2010) and interpreted according to the American College of Medical Genetics and Genomics/Association for Molecular Pathology (ACMG/AMP) guideline and relevant updates (Richards et al., 2015; Abou Tayoun et al., 2018; Ghosh et al., 2018; Biesecker et al., 2018; Brnich et al., 2019).

### Multiple sequence alignment

APOA5 protein sequences from diverse species were retrieved from the National Center for Biotechnology Information (NCBI). Multiple sequence alignment was executed using ClustalW within MEGA12 (Kumar et al., 2024). AAs frequencies were visualized using MEME Suite (Bailey et al., 2015).

### In silicon prediction for structure stability and structure change

The protein structure file of the native APOA5 was obtained from AlphaFold Protein Structure Database (Varadi et al., 2024) (https://alphafold.com/entry/AF-Q6Q788-F1). Change in stability of protein structure was assessed online using Dynamut2 (Rodrigues et al., 2021) (https://biosig.lab.uq.edu.au/dynamut2). A missense mutation was assumed to affect protein stability if the calculated Gibbs Free Energy Change <0.0 kcal/mol. The structure of mutated APOA5 was predicted online by AlphaFold 3 (Abramson et al., 2024) (https://deepmind.google/science/alphafold/alphafold-server/). Visualization of protein structure superposition was conducted online in RCSB protein Data Bank (Bittrich et al., 2024) (https://www.rcsb.org/alignment).

## Results

### Overview of the hypertriglyceridemia family

**FIGURE 1:**
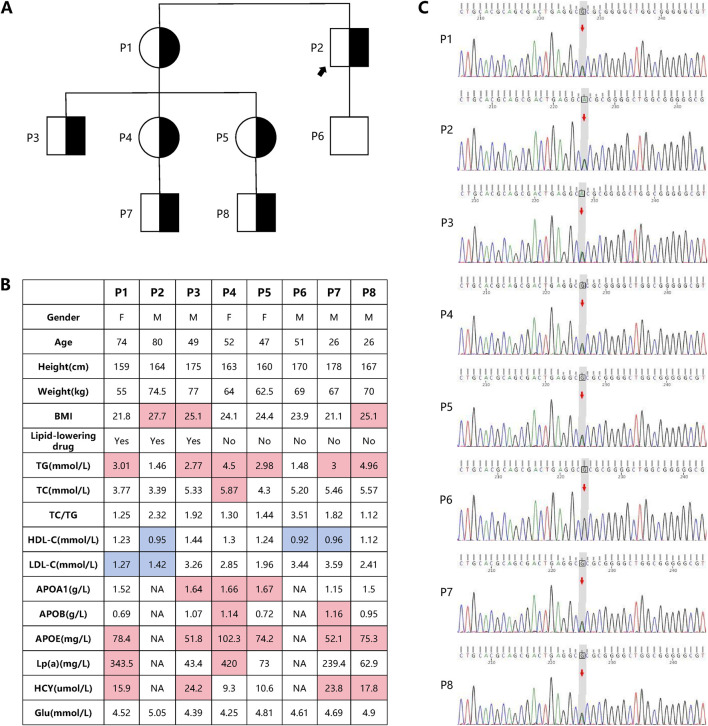
Pedigree, basic information and results of biochemical testing for lipid metabolism, and Sanger sequencing results of the HTG family. **(A)** Pedigree of the HTG family. Black arrow: proband; Half-filled symbol with black: individual who carries the heterozygous *APOA5* p. R223C mutation; Unfilled symbol: non-carrier. **(B)** Basic information and results of biochemical testing for lipid metabolism. Age: age at the time of the examination; BMI: body mass index; TG: triglyceride (reference interval: 0.56–1.71 mmol/L); TC: total cholesterol (2.50–5.70 mmol/L); HDL-C: high-density lipoprotein cholesterol (1.0–2.0 mmol/L); LDL-C: low-density lipoprotein cholesterol (1.81–4.92 mmol/L); APOA1: apolipoprotein A1 (1.0–1.6 g/L); APOB: apolipoprotein B (0.6–1.1 g/L); APOE: apolipoprotein E (27-45 mg/L); Lp(a): lipoprotein (a) (0–300 mg/L); HCY: homocysteine (4–15.4umol/L); Glu: fasting glucose (3.89–6.11 mmol/L); light red and light blue background color indicate that the value is above or below the reference interval, respectively. **(C)** Sanger sequencing chromatogram of the family. Double peak below the red arrow demonstrates the heterozygosity of the *APOA5* mutation (g.chr11:116661278G>A, p. R223C), while single peak indicates a homozygote.

The proband (Patient2, P2) (Figure 1A) was detected with elevated TG levels at age of 56 in 2001. The disorder was detected in his younger sister (P1) 13 years later. Two other family members P3 (son of P1) and P4 (oldest daughter of P1) were diagnosed with the same blood content deviation in 2015 and 2019 at ages of 39 and 47, respectively. Following the identification of abnormally high TG in a young descendant (P8, grandson of P1) during a routine physical examination in October 2024, the entire family (P1-P8, Figure 1A) sought comprehensive evaluation at the First Affiliated Hospital of Jishou University 1 month later.

All family members denied histories of diabetes, coronary heart disease, pancreatitis, endocrine diseases or autoimmune disorders. However, P2 and P6 had hypertension, and P3 and P4 exhibited low-grade fatty-liver. P2, P3 and P8 were classified as overweight based on body mass index (BMI) (Figure 1B). P3, P6 and P8 reported occasional alcohol consumption. Smoking history was reported in P6 (23 years, averaging 20 cigarettes per day) and P8 (8 years, averaging ten cigarettes per day). P1 and P3 were prescribed oral Atorvastatin (10 mg/day) for TG management. P2 had been taking fenofibrate (0.1 g*3/day) to lower blood lipid for years. No other use of drug especially those considered as secondary factors to HTG (Berglund et al., 2012) was reported. P2, P4, P6 and P7 engaged in occasional exercise. No family members reported adherence to a low-fat diet.

### Biochemical and genetic testing results

Biochemical testing confirmed substantially elevated fasting plasma TG levels (>1.7 mmol/L) in six family members (Figure 1B) with P8 exhibiting the highest level (4.96 mmol/L). P4 displayed elevated total cholesterol (TC). P2, P6 and P7 had reduced HDL-C concentrations, while P1 and P2 had subnormal LDL-C values (Figure 1B). Furthermore, elevated apolipoprotein E (APOE) levels were observed in six affected family members (Figure 1B). Liver and kidney function parameters of the family were predominantly within normal ranges (Table 1), except for elevated alanine aminotransferase (ALT), urea and creatinine (CREA), and decreased estimated glomerular filtration rate (eGFR) in P2.

**TABLE 1:** Results of biochemical testing for liver and kidney function.

Index (abbreviation)	Unit	References interval	P1	P2	P3	P4	P5	P6	P7	P8
Total protein (TP)	g/L	60–85	70.5	69.4	77.3	70.9	69.5	66.8	74.7	75.2
Albumin (ALB)	g/L	35–55	45.8	45.1	50.8	47.7	45.5	44.4	53.6	52.6
Globuli (GLO)	g/L	20–40	24.7	24.3	26.5	23.2	24	22.4	21.1	22.6
ALB/GLO	A/G	1.5–2.5	1.9	1.9	1.9	2.1	1.9	2.0	2.5	2.3
Total bile acid (TBA)	umol/L	0.0–12.0	6.2	6.2	3.4	2.4	2.5	4.3	4.1	6.7
Total bilirubin (TBIL)	umol/L	3.4–20.5	16.8	18.3	11.5	8	10.7	4.4	10.5	11.6
Direct bilirubin (DBIL)	umol/L	0–6.84	3.1	6.2	2.9	1.3	2.2	2.3	2.4	1.9
Indirect bilirubin (IBIL)	umol/L	0–18	13.7	12.1	8.6	6.7	8.5	2.1	8.1	9.7
Alanine aminotransferase (ALT)	U/L	0–40	20	47↑	29	33	33	18	31	41↑
Aspartate aminotransferase (AST)	U/L	0–40	24	33	26	25	28	15	21	25
Urea	mmol/L	2.50–7.14	5.3	7.5↑	4.8	4.5	7	4.6	3	NA
Creatinine (CREA)	umol/L	40–120	70.9	126.4↑	107.6	46	48.4	83.5	91.7	NA
Estimated glomerular filtration rate (eGFR)	ml/min/1.73m^2^	≥60	77.0	49.5↓	72.9	111.7	113.8	97.6	101.9	NA
Uric acid (UA)	umol/L	238–416	301	350	326	288	250	343	275	NA

eGFR is calculated using the CKD-EPI Creatinine Equation (2021) and the threshold eGFR <60 ml/min/1.73m^2^ indicates a diagnosis of possible chronic kidney disease according to the KDIGO 2024 Clinical Practice Guideline for the Evaluation and Management of Chronic Kidney Disease.

Whole-exome sequencing (WES) plus Sanger sequencing (Figure 1C) identified a rare heterozygous *APOA5* variant (g.chr11:116661278G>A, c.667C>T, p. R223C) in seven individuals except P6 (Figure 1A), and these p. R223C carriers were all suffered from HTG (criteria of ACMG/AMP guideline: PP1_Moderate). It should be pointed out that P2 was the first family member diagnosed with HTG, and he had been orally taking lipid-lowering drug for years which could explain the normal TG value in his biochemical testing result of this study. Thus, P6 became the only family member with both normal *APOA5* genotype and plasma TG level. The variant resides within the last exon (exon 4, encoding AAs 54–366) of *APOA5*. This missense variant was absent from ClinVar (Landrum et al., 2018), dbSNP (Phan et al., 2025) and gnomAD (Karczewski et al., 2020) before the time of this writing (PM2) but had been reported as potentially associated with HTG in two separate individuals (Jin et al., 2018; Liu et al., 2024) (PS4_Supporting). Variant pathogenicity prediction tools SIFT (Kumar et al., 2009), PolyPhen-2 (Adzhubei et al., 2010) and MutationTaster (Schwarz et al., 2010) rated p. R223C as “Deleterious”, “Probably damaging” and “Disease causing”, respectively. The REVEL score for this variant was 0.702 (PP3) (Pejaver et al., 2022). Taking together (PP1_Moderate + PM2+PS4_Supporting + PP3), p. R223C was classified as Likely Pathogenic (LP) according to the ACMG/AMP guideline. No other rare variants share by affected individuals were detected within a panel of TG-related genes (Jin et al., 2018) (*LPL*, *LMF1*, *APOC2*, *GPIHBP1*, *GCKR*, *ANGPTL3*, *APOB*, *APOA1*, *APOA4*, *APOC3*, *APOA5*, *TRIB1*, *CETP*, *APOE* and *LIPI*). All other shared variants were common with allele frequency (AF) > 20% (excluding intronic ones, these variants were listed in Table 2). With the exception of p. R223C, the remaining seven variants across *APOB*, *LMF1*, and *LIPI* were all classified as “Benign” or “Likely Benign” in ClinVar.

**TABLE 2:** Exonic or splicing variants among 15 TG-related genes shared by affected individuals.

P1	P3	P4	P5	P7	P8	Gene	Variant	Classification	dbSNP	gnomAD
het	het	hom	het	het	het	*APOB*	c.8216C>T (p.P2739L)	B/LB	rs676210	0.7304
hom	hom	hom	hom	hom	hom		c.6937A>G (p.I2313V)	B	rs584542	0.9981
hom	hom	hom	hom	hom	hom		c.4265A>G (p.Y1422C)	B	rs568413	1
hom	hom	hom	het	hom	hom		c.1853C>T (p.A618V)	B/LB	rs679899	0.8453
het	het	het	het	het	het	*APOA5*	c.667C>T (p.R223C)	LP	​	​
het	hom	hom	hom	het	het	*LMF1*	c.756G>A (p.A252A)	B	rs2076425	0.249
hom	hom	hom	hom	hom	hom		c.729 + 18C>G	B	rs11864203	0.5336
hom	hom	hom	hom	hom	hom	*LIPI*	c.1263A>T (p.R421S)	B	rs7283442	1

Het: heterozygous; Hom: homozygous; B: benign; LB: likely benign; LP: likely pathogenic; gnomAD, uses the frequency data of east Asian population.

HTG-related risk variants were also investigated through six affected individuals (P2 and P6 did not received WES testing). Variants reported to be associated with elevated TG levels in Chinese population with at least three literature evidence were manually curated (Table 3). Each of the six individuals carried one or two of these variants. Specifically, *GCKR* c.1337 T>C (p.L446P) variant (rs1260326) (Yuan et al., 2022; Shen et al., 2013; Lu et al., 2017) was detected in five individuals (P1, P4, P5, P7 and P8). The missense variant was common in Chinese population with AF of 49.94%. Another common variant, *APOA5* c.-3A>G (rs651821) (You et al., 2018; Zhu et al., 2014; Li Y. et al., 2023; Wang et al., 2020; Tan et al., 2012; Liu et al., 2010; Zhou et al., 2013) located in 5′untranslated regions (UTR), was discovered in P3, P4 and P5 in heterozygous manner. Additionally, risk variant rs2075291 (Lu et al., 2017; Liu et al., 2010; Tang et al., 2006; Liu et al., 2025; Qian et al., 2018; Li et al., 2011) was only detected in P8, and rs662799 (You et al., 2018; Zhu et al., 2014; Liu et al., 2010; Jiang et al., 2010; Ong et al., 2011; Xu et al., 2013) and rs2266788 (Liu et al., 2010; Cui et al., 2014; Shou et al., 2014) were not detected in any of the six family members.

**TABLE 3:** Risk variants closely associated with elevated TG levels in Chinese population.

P1	P3	P4	P5	P7	P8	Gene	Variant	dbSNP	gnomAD
-	-	-	-	-	-	*APOA5*	−1131 T>C	rs662799	0.6712
-	-	-	-	-	het		c.553G>T (p.G185C)	rs2075291	0.0789
-	het	het	het	-	-		c.-3A>G	rs651821	0.7121
-	-	-	-	-	-		c.*158C>T	rs2266788	0.7485
het	-	hom	het	het	hom	*GCKR*	c.1337 T>C (p.L446P)	rs1260326	0.4994

gnomAD uses the frequency data of east Asian population. The variant APOA5 c.*158C>T is also referred to as “c.1891T>C”.

### Residue conservation analysis of APOA5 position 223

**FIGURE 2:**
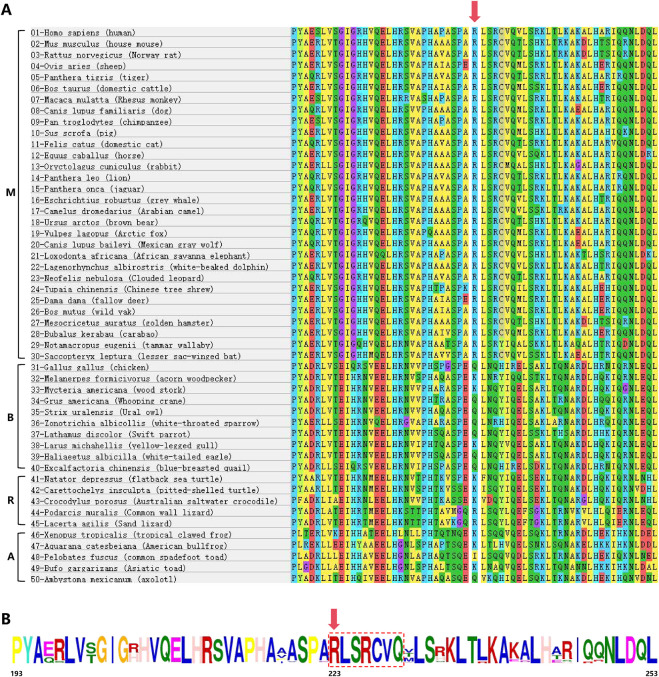
Multiple sequence alignment and AA frequency visualization of the APOA5 protein sequences. **(A)** Multiple sequence alignment result of the APOA5 protein sequences from 50 species. Red arrow: the position 223; (M) mammal; (B) bird; (R) reptile; (A) amphibians; **(B)** The frequencies of the AAs of the position 223 and flanking residues (±30) across the 30 mammals. Red dashed box: a highly conserved heptapeptide.

Strictly conserved residues across species often indicate critical importance for protein structure and function (Valdar, 2002). To investigate the conservation of residue 223 in APOA5, a comprehensive multiple sequence alignment was performed using sequences from 30 mammals (Figure 2A, Rows 1–30), 10 birds (Rows 31–40), 5 reptiles (Rows 41–45), and 5 amphibians (Rows 46–50). The mammalian cohort included common species (e.g., house mouse, sheep, cattle, dog, pig, cat, horse, rabbit), region-specific species (e.g., Arabian camel, Arctic fox, Mexican gray wolf, African savanna elephant, Chinese tree shrew), marine mammals (grey whale, white-beaked dolphin), and a flying mammal (lesser sac-winged bat). The alignment revealed strict conservation of arginine (R) at position 223 across all 30 mammalian species (Figure 2A, red arrow). This profound evolutionary conservation implies that substitutions at this site likely impair APOA5 structure and function in mammals, including humans. Birds (Rows 31–40) exhibited glutamine (Q) or lysine (K) at this position. In reptiles (Rows 41–45), of note, two kinds of lizard utilized the same AA (arginine, R) as mammals, while turtle and crocodile used lysine (K) which was also preferred by three amphibians in this alignment. Common spadefoot toad employed a unique isoleucine (I) at position 223.

Amino acid frequencies at position 223 and flanking residues (±30) across the 30 mammalian (not the whole 50 species) are displayed in Figure 2B. AAs in position 223 and the following six positions were invariant among mammals. The conserved heptapeptide motif RLSRCVQ (Figure 2B, red dashed box) suggests functional significance.

### Impact of p.R223C on APOA5 structural stability

**FIGURE 3:**
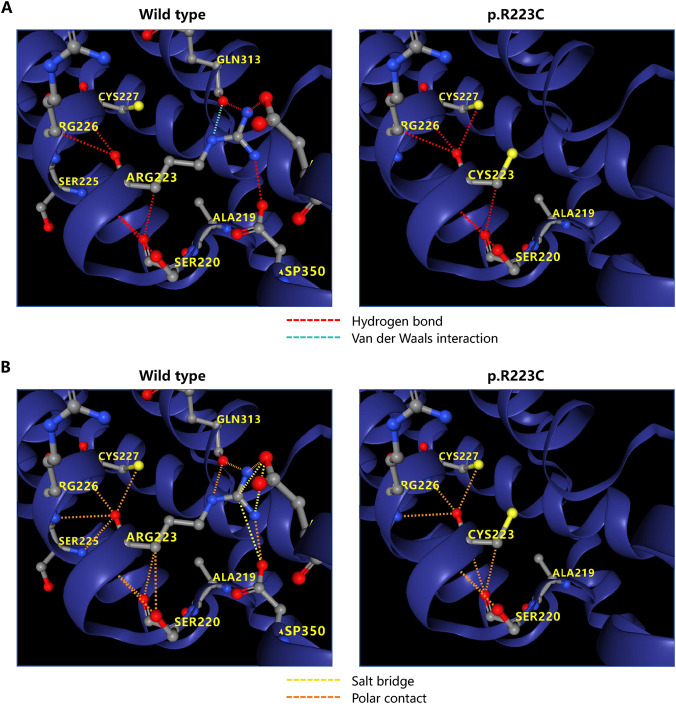
The predicted residue interaction states of the position 223 of the wild-type (left) and the mutated APOA5 protein (right) from the perspective of **(A)** Hydrogen bond and Van der Waals interaction, and **(B)** salt bridge and polar contact. Hydrogen bond, Van der Waals interaction, salt bridge and polar contact are denoted as red, light blue, yellow and orange dashed line, respectively.

Gibbs Free Energy change (ΔΔG) was introduced to measure the predicted stability change and a missense mutation was presumed to undermine protein stability if ΔΔG<0.0 kcal/mol. The p. R223C mutation resulted in the loss of hydrogen bonds (Figure 3A, red dashed line) as well as a Van der Waals interaction (Figure 3A, light blue dashed line) between the guanidyl of the native arginine and neighboring residues (Q313, D346 and D350). Furthermore, replacing the positively charged arginine with uncharged cysteine abolished ionic bonds also known as salt bridges (Figure 3B, yellow dashed lines) between residue 223 and D346/D350. The polar contact network surrounding residue 223 was also partially disrupted, with five polar contacts lost (Figure 3B, orange dashed lines). The predicted ΔΔG was −0.61 kcal/mol, supporting the hypothesis that p. R223C destabilizes the APOA5 structure.

### Short germline *APOA5* variants in ClinVar

ClinVar is a website which collects and displays genetic variants data submitted by researchers and physicians around the world. During the time of this writing, we had submitted information about our finding on the p. R223C variant to ClinVar (https://www.ncbi.nlm.nih.gov/clinvar/variation/4075749/?term=SCV006308021). And there were totally 15 short germline *APOA5* variants deposited in the website including ours (Table 4). These variants were all classified as pathogenic (P) or likely pathogenic (LP). Except two with unprovided condition, the rest were all involved with cardiovascular disorders such as HTG and hyperlipoproteinemia (HLP). Besides, our submission provided the only missense variant. Other five frameshift variants and nine nonsense variants (Table 4) all introduced premature translation termination, resulting in protein truncation in varying degrees (Supplementary Material S1).

**TABLE 4:** Short germline *APOA5* variants (<50 basepairs) collected by ClinVar.

Exon	cDNA change	Protein change	Type (consequence)	Condition	Classification
2	c.42dup	L15fs	Duplication (frameshift)	CV	**P**
	c.117_120del	R40fs	Deletion (frameshift)	HLP type 5	LP
3	c.154G>T	E52*	SNV (nonsense)	CV	LP
4	c.289C>T	Q97*	SNV (nonsense)	HLP type 5, HTG, CV	P
	c.415C>T	Q139*	SNV (nonsense)	HLP type 5	P
	c.466G>T	E156*	SNV (nonsense)	CV	LP
	c.562A>T	K188*	SNV (nonsense)	CV	P
	c.579_592del	Y194fs	Deletion (frameshift)	HLP type 5	P
	c.667C>T	R223C	SNV (missense)	HTG	LP
	c.681C>A	C227*	SNV (nonsense)	HTG	LP
	c.775A>T	R259*	SNV (nonsense)	CV	LP
	c.795del	T266fs	Deletion (frameshift)	CV	LP
	c.847C>T	Q283*	SNV (nonsense)	not provided	LP
	c.883C>T	Q295*	SNV (nonsense)	not provided	P
	c.990_993del	D332fs	Microsatellite (frameshift)	HLP type 5, HLP type 4, CV	P/LP

SNV: single nucleotide variant; CV: cardiovascular phenotype; HLP: hyperlipoproteinemia; P: pathogenic; LP: Likely pathogenic.

## Discussion

*APOA5* was discovered in 2001 b y comparative genomics and the plasma TG of knockout mice lacking *Apoa5* was found four times higher than controls (Pennacchio et al., 2001). Subsequent studies confirmed the association between APOA5 deficiency and elevated plasma TG (Yang et al., 2024), although the underlying mechanism was not elucidated clearly for years. Chen et al. proposed that APOA5 bind to the angiopoietin-like protein 3/8 complex (ANGPTL3/8), suppressing its capacity to inhibit LPL catalytic activity (Chen et al., 2021). They suggested that APOA5 lowered TG was via the competition with LPL for the same ANGPTL3/8-binding site (Balasubramaniam et al., 2022). Consequently, APOA5 deficiency could impede LPL’s hydrolyzation on TG in lipoproteins and hence promoted the TG accumulation in plasma. Subsequent research from the same group identified a region within the APOA5 carboxyl-terminus as essential for inhibiting ANGPTL3/8 activity (Chen et al., 2024). According to our *in silico* prediction, the p. R223C mutation leaded to possible polarity alteration (Figure 3B) and discernible structure change (Supplementary Material S1). However, the absence of a resolved ANGPTL3/8 complex structure (e.g., via cryo-EM or X-ray crystallography) currently precludes prediction of whether or how the p. R223C mutation identified here might affect APOA5-ANGPTL3/8 binding.

The wild-type APOA5 structure was derived from the AlphaFold Protein Structure Database. Although the predicted local confidence (pLDDT) for residue 223 and its immediate vicinity is high, other regions, particularly the N- and C-termini, exhibit lower confidence scores (https://alphafold.com/entry/Q6Q788). While artificial intelligence has revolutionized protein structure prediction and bring much convenience to scientists in the field, the reliability of predicted results necessitates careful consideration.

Due to economic constraints, P2 and P6 did not undergo WES testing. Nevertheless, the Sanger sequencing results of the two (Figure 1C) are sufficient for supporting the finding of this study. All eight family members underwent comprehensive biochemical profiling, including assessments of lipid metabolism (Figure 1B) and liver/kidney function (Table 1). The latter is essential to exclude hepatic or renal dysfunction as primary causes of dyslipidemia before attributing abnormal TG levels to genetic factors. Notably, APOE levels were elevated in six affected individuals (Figure 1B). While a previous research revealed that accumulation of APOE might contribute to HTG (Huang et al., 1998), no shared *APOE* variant was identified within the six (Table 2). A potential link between the p. R223C variant and elevated APOE needs further investigation.

As mentioned before, the manifestation of HTG might originate from either monogenic or polygenic basis, and could be exacerbate by concomitant environmental factors. In this study, P8 exhibited the highest TG concentration (4.96 mmol/L), possibly attributable to the combined effects of the p. R223C variant, the two co-occurring HTG-risk variant (less common rs2075291 and homozygous rs1260326), and lifestyle (smoking and alcohol consumption). Another five affected individuals in the family also carried one or two risk variants (Table 3), and some of them also had conditions categorized as non-genetic etiology to HTG. Hence, even though p. R223C was classified as LP according to the principles in ACMG/AMP guideline, to what extent this variant alone could increase plasma TG, needs to be further confirmed by well-established *in vitro* or *in vivo* functional studies.

Totally four *APOA5* variants reported to be closely involved with HTG in Chinese population were manually curated in this study (Table 3). Specifically, rs662799 (−1131 T>C) (Martinelli et al., 2007; Maasz et al., 2007; Sousa et al., 2008; Ariza et al., 2010; de Luis et al., 2021), rs651821 (c.-3A>G) (Iannuzzi et al., 2022; Tabb et al., 2017) and rs2266788 (c.*158C>T) (Caussy et al., 2014; Sumegi et al., 2017; Horvatovich et al., 2011) were also found to be related to HTG in Europeans. It is worth noting that these three are all non-coding variants located in promoter region, 5′UTR and 3′UTR separately, with high AF (>60%) across all population according to the information in dbSNP. The remaining rs2075291 (c.553G>T, p. G185C) was recognized as a HTG risk variant among East Asians (Matsunaga et al., 2020; Kim et al., 2017; Huba and cek, 2016) (AF = 7%, vs. 0.03% in Europeans). In addition, our literature search showed that a HTG-associated *APOA5* variant rs3135506 (c.56C>G, p. S19W), existing in Europeans (Ariza et al., 2010; Dallongeville et al., 2006; Henneman et al., 2007; Wang et al., 2007) (AF = 5.7%, vs. 0.2% in East Asians), Tunisians (Kefi et al., 2017) and African Americans (Jiang et al., 2024), was hardly reported in Chinese or East Asians. This is in consistent with a previous summary on *APOA5* (Huba and cek, 2016). Hence, the above two coding variants exhibit distinct population specificity.

The coding variant p. R223C was initially discovered in a single Chinese case in 2024 (Liu et al., 2024) and then reported in a Chinese family in this study. It is easy to associate it with another possibly population-specific *APOA5* variant for HTG. However, this variant is absent from dbSNP and gnomAD, and the AF is as low as 0.007% in the Han Chinese Genomes Database (PGG.Han) (Gao et al., 2020) which archives genomic data of more than ten thousand Chinese individuals. The few reports is currently not enough to strongly support the speculation that p. R223C is population-specific. Nevertheless, it is believed that p. R223C will receive more attention in the future after the publication of family-based evidence collected in this study, which is beneficial to further understanding the population specificity and disease penetrance of the variant.

P2 was the first family member diagnosed with HTG in the year of 2001, however we had already lost the specific TG value of the patient at that time. He had been taking fenofibrate for many years and this agent demonstrated a significant therapeutic effect to reduce TG (Figure 1B). Thus we had not adjusted his therapeutic regimen even though fenofibrate is normally prescribed for individuals with abnormally high TG levels. Notably, the eGFR value (Table 1) suggested a possible chronic kidney disease in P2 according to the KDIGO 2024 Clinical Practice Guideline for the Evaluation and Management of Chronic Kidney Disease (Kidney Disease: Improving Global Outcomes (KDIGO) CKD Work Group, 2024), and we are now considering to modify the medication regimen for the patient because the metabolism and excretion of fenofibrate are highly dependent on renal function. Both P1 and P3 had elevated level of TG, TC and LDL-C before treatment, and were prescribed atorvastatin according to the 2023 Chinese Guideline for Lipid Management (Li JJ. et al., 2023) which pointed out that statins were indicated for the treatment of mixed hyperlipidemia. P4, P5, P7 and P8 had not taken any medication to control lipid level for reasons including insufficient awareness of the hazards of HTG, aversion to oral medications, and no obvious impairment upon quality of life caused by the disorder. At present, all the family memebers are receiving dietary therapy. Although the existing evidence (Hubacek et al., 2009; Bogari et al., 2019; Lai et al., 2007; Perez-Martinez et al., 2009; Brautbar et al., 2011; Brautbar et al., 2013; Liu et al., 2009) clearly shows that specific *APOA5* variants are important factors affecting the efficacy of fenofibrate and atorvastatin, we have not yet explored whether the p. R223C variant affects the efficacy of both agents on TG levels because of the limited cases and funds. This is a potential research direction that we will focus on in the future.

WES was utilized in this study for genetic testing. However, this technology has limitations in detection of variants too far away from coding regions, copy number variants (CNV) especially those ≤ 100 kb and structural variants (SV). It could be one of the potential reasons for the non-detection of rs662799 in regulatory region and rs2266788 in 3′UTR (Table 3).

## Conclusion

Inferring novel HTG-associated variants based solely on isolated cases poses challenges for clinical reliability. In this study, seven HTG-affected family members carried the rare *APOA5* variant p. R223C in heterozygous manner, while the rest one of the family was a non-carrier with normal plasma TG level. This association of genotype and phenotype within a family provides valuable evidence supporting the probable deleteriousness of the p. R223C variant in moderate HTG, offering insights for molecular diagnosis and mechanism research on HTG caused by APOA5 deficiency.

## Data Availability

The original contributions presented in the study are included in the article/Supplementary Material, further inquiries can be directed to the corresponding authors.
