# Supplementary material for: Molecular characterization of a rare heterozygous APOA5 variant in a Chinese family with moderate hypertriglyceridemia
Source: Front Genet. 2026 Jun 2;17:1796970. doi: 10.3389/fgene.2026.1796970 (PMC13268604; doi:10.3389/fgene.2026.1796970)
Supplement: Supplementary file 1 [file Supplementaryfile1.docx]

**Supplementary File 1 Visualization of protein structure superposition of the native (brown) and mutated (blue) APOA5 protein.**

**
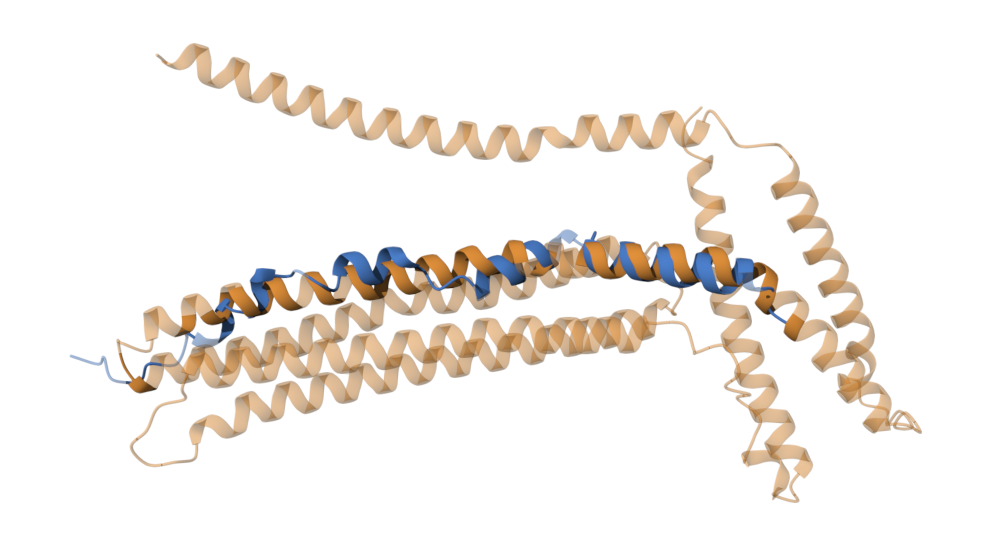
**

C-termini

**L15fs**

**
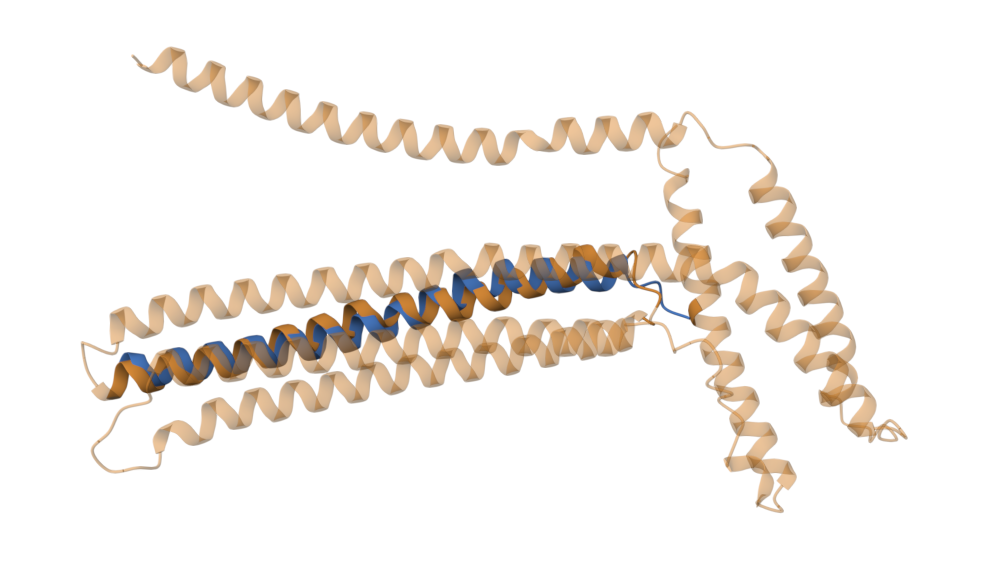
R40fs
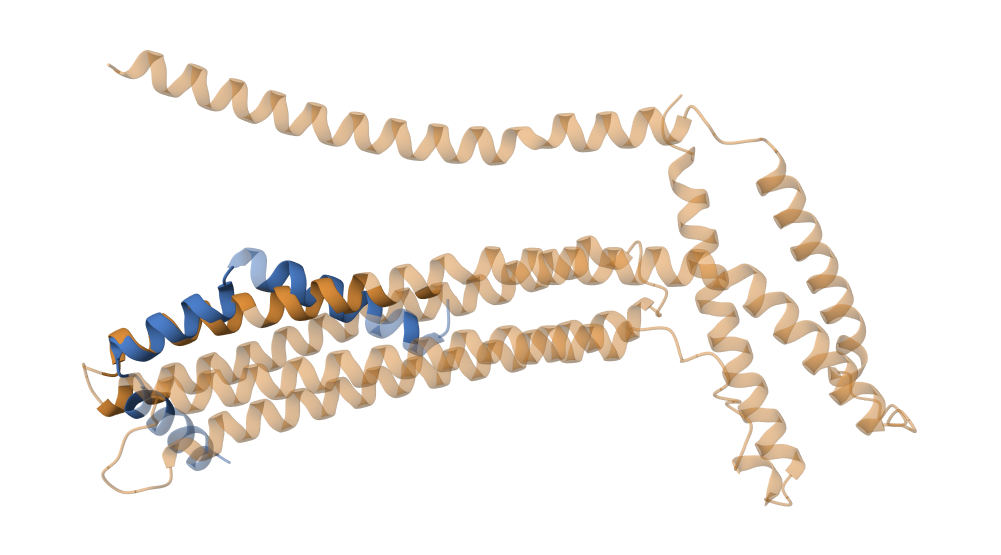
E52***

**
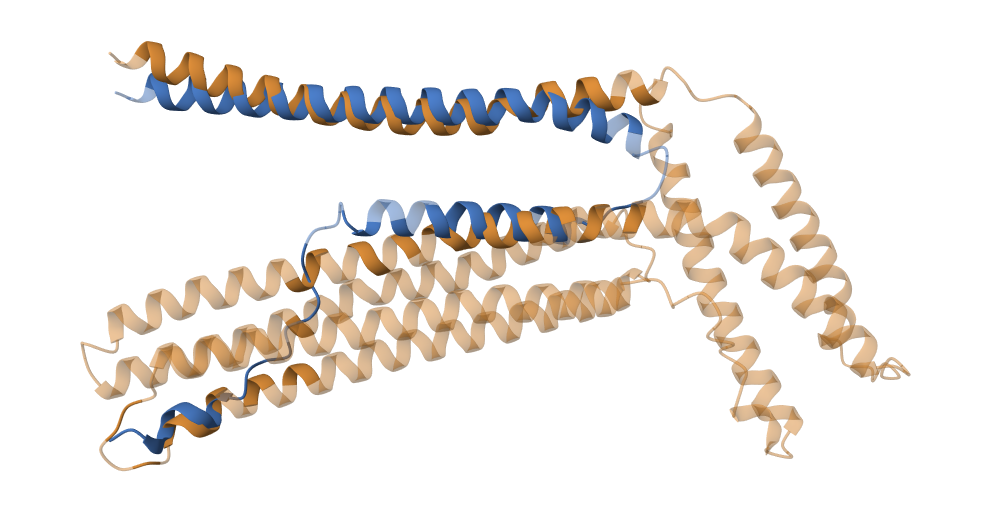
Q97***

**
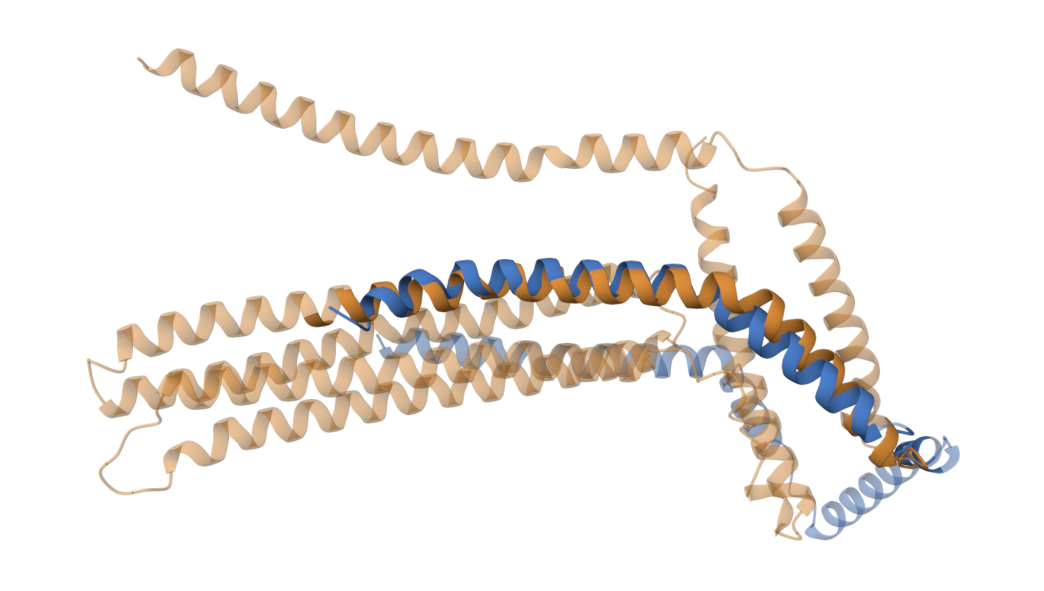
Q139***

**
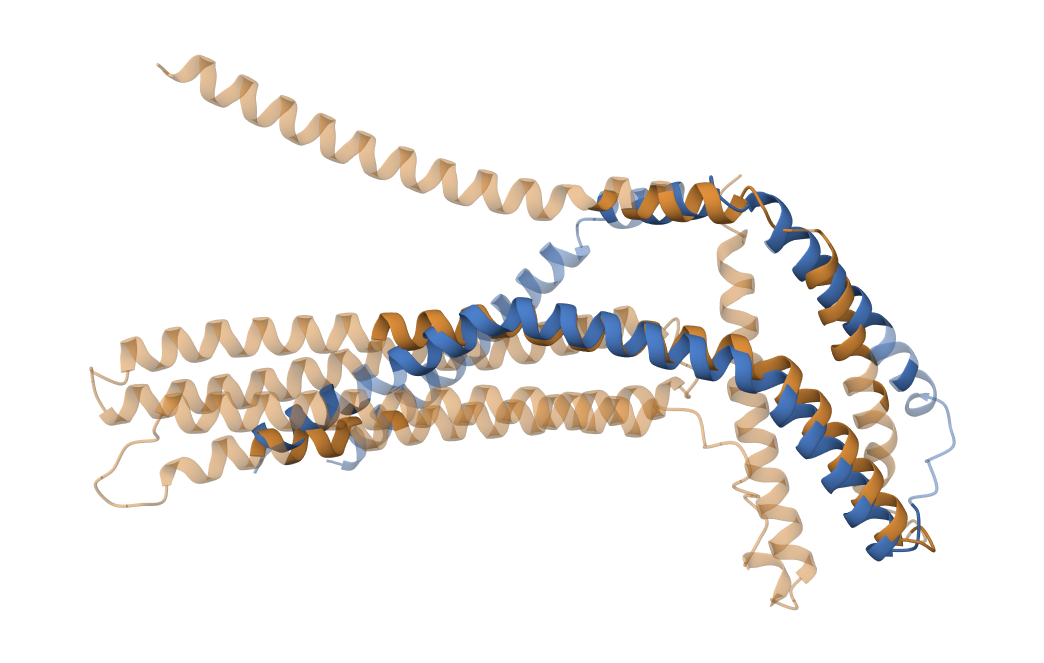
E156*
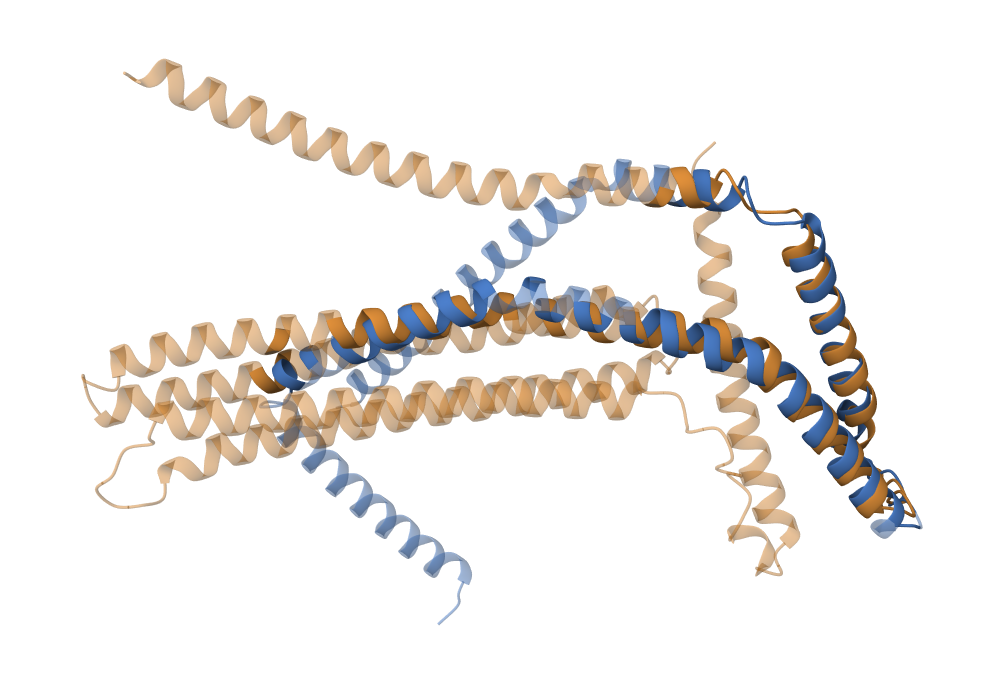
K188***

**
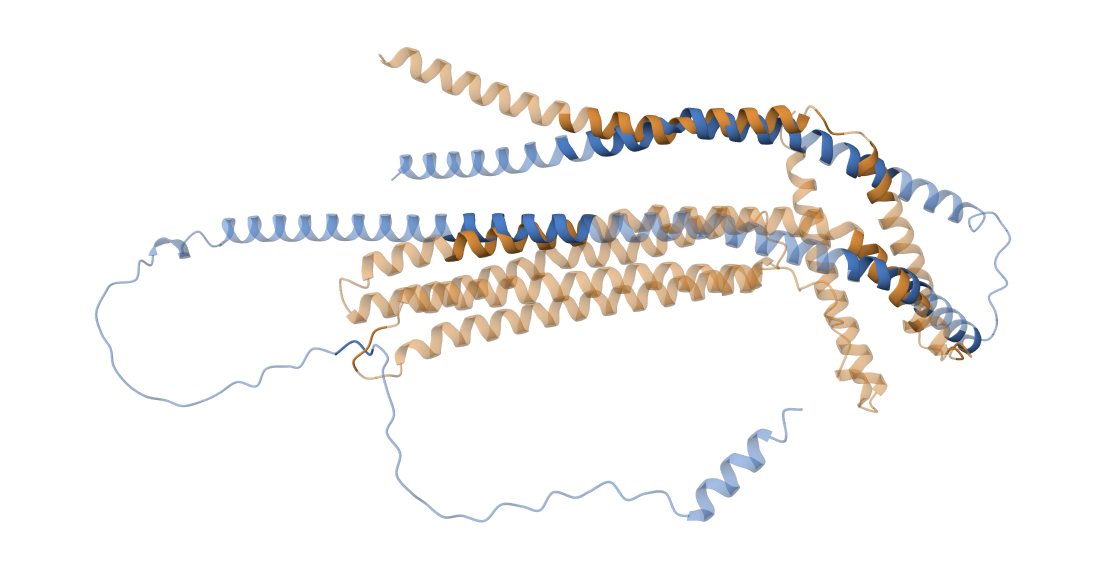
Y194fs
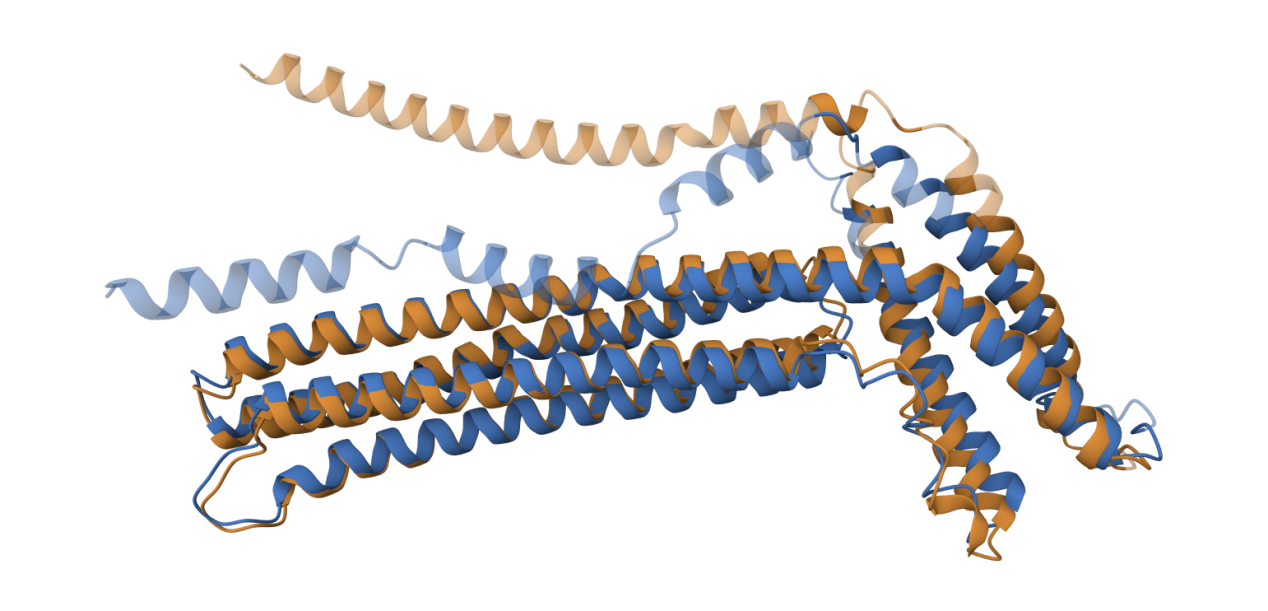
R223C**

**
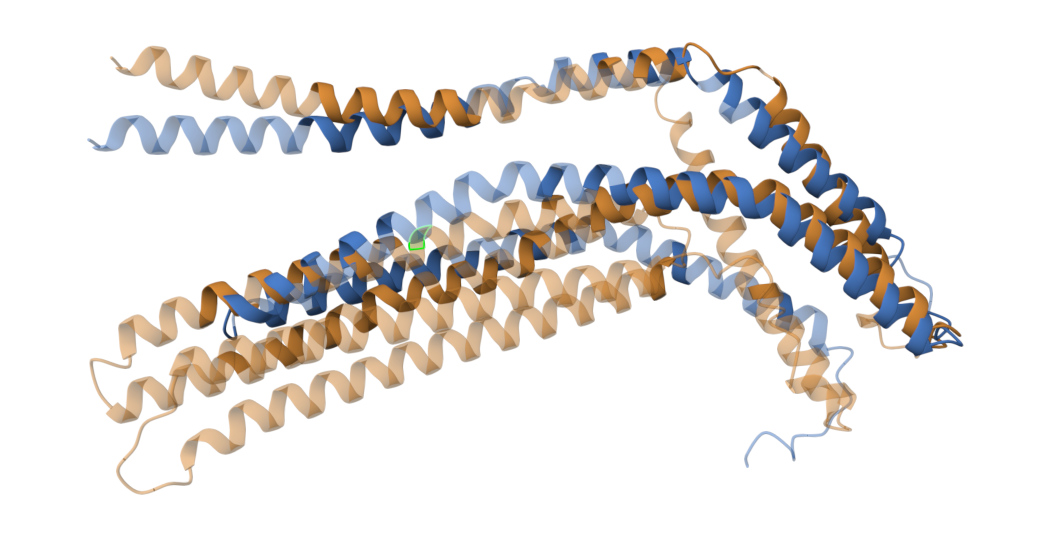
C227*
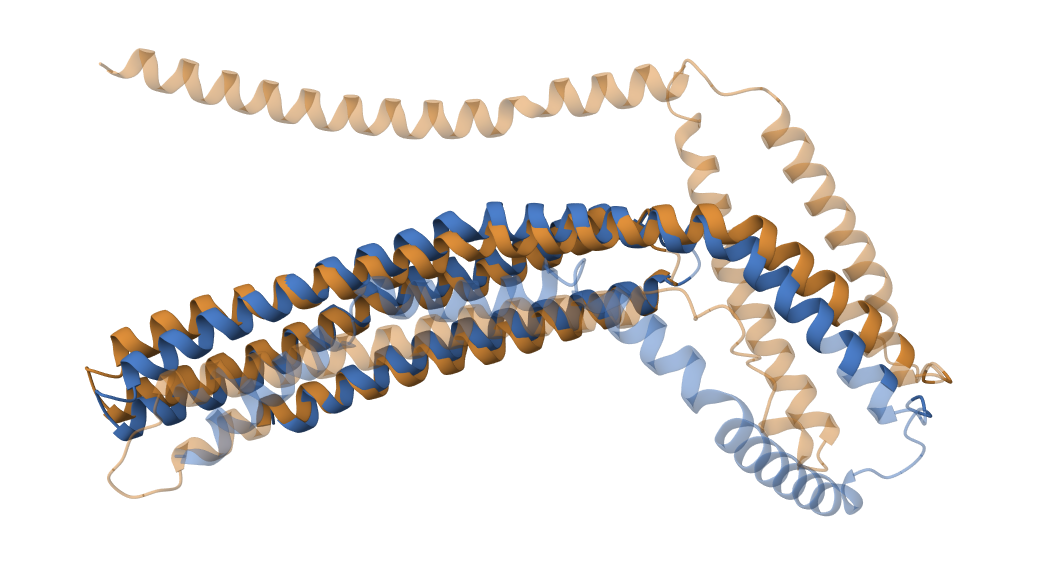
R259***

**
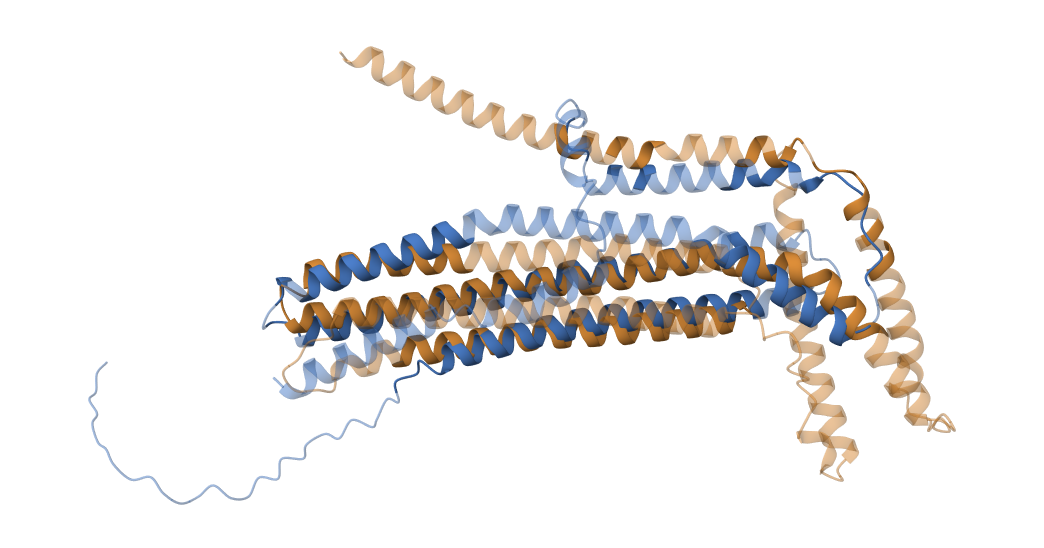
T266fs
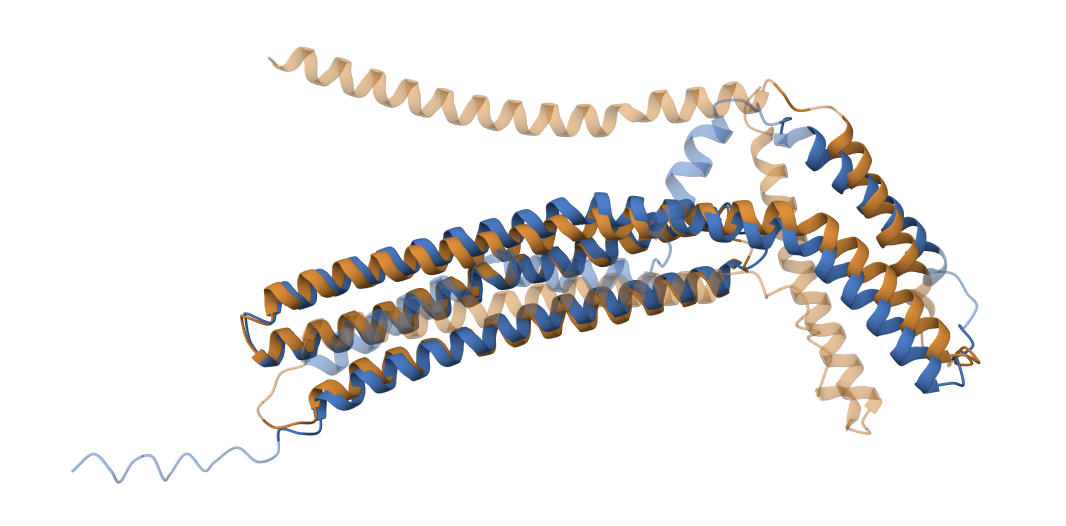
Q283***

**
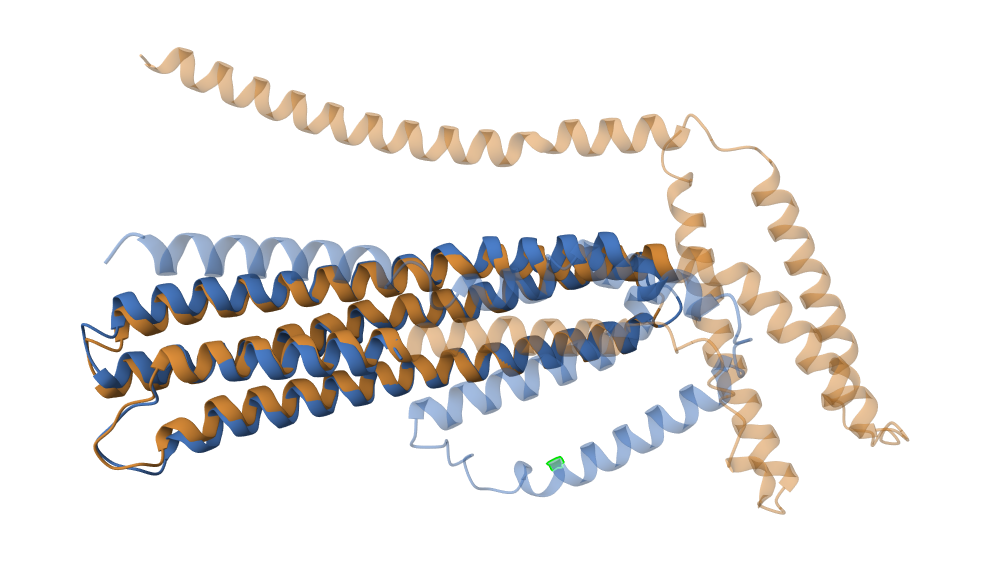
Q295*
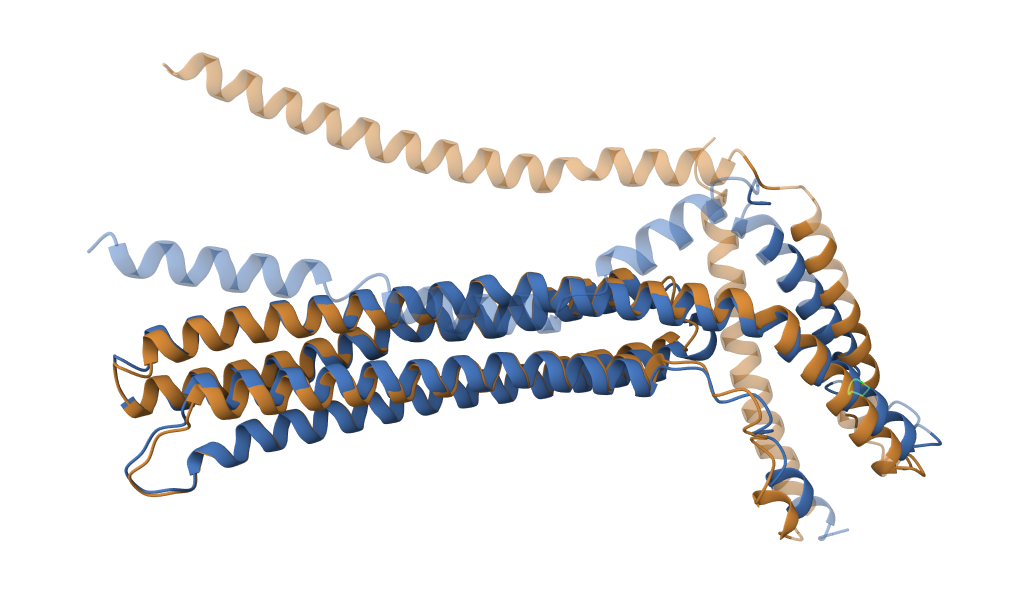
D332fs**
